# CSE1L participates in regulating cell mitosis in human seminoma

**DOI:** 10.1111/cpr.12549

**Published:** 2018-11-28

**Authors:** Chunyan Liu, Jiajing Wei, Kang Xu, Xiaosong Sun, Huiping Zhang, Chengliang Xiong

**Affiliations:** ^1^ Family Planning Research Institute, Tongji Medical College Huazhong University of Science and Technology Wuhan China; ^2^ The First People’s Hospital of Tianmen City Tianmen China; ^3^ Xiangyang Central Hospital, Affiliated Hospital of Hubei University of Arts and Science Xiangyang China; ^4^ Wuhan Tongji Reproductive Medicine Hospital Wuhan Hubei China

**Keywords:** cell mitosis, cell proliferation, CSE1L, seminoma, spindle

## Abstract

**Objectives:**

CSE1L has been reported to be highly expressed in various tumours. Testicular germ cell tumours are common among young males, and seminoma is the major type. However, whether CSE1L has functions in the seminoma is unclear.

**Materials and methods:**

The expression of CSE1L was detected by immunohistochemistry in seminoma tissues and non‐tumour normal testis tissues from patients. CSE1L distribution during cell mitosis was determined by immunofluorescent staining with CSE1L, α‐tubulin and γ‐tubulin antibodies. The effects of Cse1L knockdown on cell proliferation and cell cycle progression were determined by Cell Counting Kit‐8 assay, flow cytometry, PH3 staining and bromodeoxyuridine incorporation assay.

**Results:**

CSE1L was significantly enriched in the seminoma tissue compared with the non‐tumour normal testis tissue. CSE1L also co‐localized with α‐tubulin in the cells with a potential to divide. In the seminoma cell line TCam‐2, CSE1L was associated with the spindles and the centrosomes during cell division. The knockdown of CSE1L in TCam‐2 cells attenuated the cells’ proliferative capacity. Cell cycle assay revealed that the CSE1L‐deficient cells were mainly arrested in the G0/G1 phase and moderately delayed in the G2/M phase. The proportion of cells with multipolar spindle and abnormal spindle geometry was obviously increased by CSE1L expression silencing in the TCam‐2 cells.

**Conclusions:**

Overall, these findings showed that CSE1L plays a pivotal role in maintaining cell proliferation and cell division in seminomas.

## 1 INTRODUCTION

Testicular germ cell tumours (TGCTs) are the most common cancers in young males.^1^, ^2^ These tumours can be distinguished into two major histological subtypes: seminomas and non‐seminomas. Seminomas are the most common cancer type of TGCTs.^3^, ^4^ Although TGCT is a rare cancer, its incidence has increased with years, whereas most other malignancies have stabilized or declined in emergence over the past years.^5^ The aetiology of TGCT is also not well investigated and remains unclear.

The cellular apoptosis susceptibility gene (CAS, also named CSE1L) encodes a protein of about 100 kDa in molecular mass that distributes in the nuclei and cytoplasm of cells; the protein was first isolated in MCF‐7 breast cancer cells subjected to *Pseudomonas* exotoxin‐induced apoptosis.^6^ Then, CSE1L has been found to play multiple roles in cellular functions, including cell proliferation,^7^, ^8^ apoptosis,^9^ microvesicle formation,^10^ nucleocytoplasmic transport,^11^ epigenetic silencing^12^ and embryonic development.^13^, ^14^ CSE1L also functionally interacts with P53 and associates with a panel of P53 target gene promoters to determine cellular outcome.^15^

The CSE1L gene maps to 20q13, a chromosome region correlated with the development of solid tumours.^16^ CSE1L is highly expressed in various types of cancers, such as ovarian tumours,^17^ hepatocellular carcinoma (HCC),^7^ lymphomas,^18^ colorectal tumours,^19^ breast tumours,^8^ melanomas,^20^ bladder cancer,^21^ lung cancer,^22^ oligodendroglial tumours^23^ and thyroid tumours,^24^ and CSE1L expression is correlated with cancer grade, cancer stage and poor cancer outcome.^25^ However, the regulatory mechanism of the CSE1L signalling pathway on cancer progression is still obscure; only a few studies have reported on the interaction of CSE1L with other cancer signalling pathways. In ovarian cancer cell lines, CSE1L regulates the expression of the pro‐apoptotic genes RASSF1C and RASSF1A to protect tumour cells from death.^26^ Another study suggested that AKT activation forces the nuclear accumulation of CSE1L in the ovarian cancer cell, likely to induce pro‐oncogenic signals.^17^ Winkler et al^27^ demonstrated that CSE1L and its transport substrate importin‐α1 (imp‐α1) are highly expressed in HCC and maintain HCC cell survival by regulating the X‐linked inhibitor of apoptosis. In melanogenesis, CSE1L links and regulates cAMP/PKA and Ras/ERK signal pathways to induce CREB and MITF expression.^28^ In another study, the CSE1L protein was found to interact with mutS homolog 6 (MSH6) and positively regulate the MSH6 protein to promote osteosarcoma progression.^29^ CSE1L, also as a microvesicle membrane protein, can be detected in tumour‐derived exosomes/microvesicles.^10^ Because tumour cells secrete exosomes/microvesicles more frequently than do normal cells, CSE1L can be used as a diagnostic marker for tumours.^30^

Despite all these functions of CSE1L reported in multiple types of cancers, the clinical significance of CSE1L in testicular cancer has not been demonstrated. Herein, we found that the CSE1L protein is enriched in human seminoma tissue samples. We further knocked down CSE1L in a seminoma cell line TCam‐2 to investigate CSE1L function in testicular cancers. We also utilized immunofluorescence images to show how CSE1L is associated with mitotic spindles during the TCam‐2 cell cycle and may facilitate seminoma cell division.

## 2 MATERIALS AND METHODS

### 2.1 Human tissue and cell culture

The study protocol was approved by the Ethics Committee of the Xiangyang Central Hospital (approval ID: 2017‐004) and The First People’ Hospital of Tianmen City (approval ID: (2017)3). Informed consent was obtained from each participant. All procedures were conducted in line with the principles of the Declaration of Helsinki and the relevant guidelines.

Human testis and seminoma tissue from four patients were obtained from the Xiangyang Central Hospital and The First People’ Hospital of Tianmen City and fixed in Bouin's solution (Sigma, Munich, Germany). The samples were then embedded in a solid block of paraffin wax for use in permanent slides.

TCam‐2 cells were cultured in high‐glucose DMEM media (Gibco, USA) supplemented with 10% foetal bovine serum (FBS; Gibco, Grand Island, NY, USA) at 37°C and 5% CO_2_ incubation.

### 2.2 Immunohistochemistry and immunocytochemistry

The seminoma tissues embedded in paraffin were sectioned to 4 μm slices and mounted to slides. Slides were gradient dewaxed, rehydrated and processed for heat‐activated antigen retrieval by using a microwave for 10 minutes. Then, the slides were cooled to room temperature. For immunofluorescence, the slides were blocked and then incubated with rabbit anti‐CSE1L antibody (1:100; Abcam, Cambridge, UK), mouse anti‐PCNA antibody (1:200; Abcam) and mouse anti‐α‐tubulin (1:200; Sigma) overnight at 4°C. After PBST washing for three times, the sections were probed with Alexa Fluor 488‐labelled donkey anti‐rabbit IgG (H + L; 1:200; ZSGB‐BIO, Beijing, China) and Alexa Fluor 594‐labelled donkey anti‐mouse IgG (H + L; 1:200; ZSGB‐BIO) for 2 hours in the darkness at room temperature. For immunohistochemical staining, the slides were conducted in accordance with the manufacturer's protocol for the ChemMate™ DAKO EnVision™ detection kit (Dako, Hamburg, Germany). For cell slide preparation, one round cover slide was plated into each well of a six‐well plate, and the cells were seeded evenly across the wells. After the cells were 70% confluent, the slides were fixed with 4% PFA for 15 minutes and washed with cold PBS for 5 minutes. After blocking, the cell slides were incubated with primary antibodies, including CSE1L (1:200; Abcam), α‐tubulin (1:500; Sigma), γ‐tubulin (1:200; Sigma) and serine‐10‐phosphorylated histone H3 (PH3; 1:500; CST, Boston, MA, USA). Fluorescence‐labelled secondary antibodies (ZSGB‐BIO) were used at 1:200 dilutions, and cell nuclei were counterstained with DAPI. Images were captured under a confocal laser‐scanning microscope (Leica, Nussloch, Germany) and an Olympus microscope.

### 2.3 IHC data analysis

Average staining of CSE1L protein in seminoma tissue and non‐tumoral normal testis tissue was quantified by colour deconvolution of IHC micrographs using the IHC profiler plugin integrated into the ImageJ software.^31^ Five fields per IHC slide were calculated, and the most frequent score was selected as the score result of this slide; IHC score: 3+ (highly positive); 2+ (positive); 1+ (low‐positive); 0 (negative). Eight slides of IHC experiments were performed on each tissue sample and quantified by the above method. During the evaluation, IHC slides were blind‐coded.

### 2.4 RNA interference (RNAI)

Pre‐designed siRNA sequences targeted to human CSE1L (KD1: 5ʹ‐GCATGATCCTGTAGGTCAA‐3ʹ; KD2: 5ʹ‐GCATGGAATTACACAAGCAAA‐3ʹ; KD3: 5ʹ‐GCAGTTAAGTGATGCAATT‐3ʹ) were synthesized by the GenePharma Company and transfected into TCam‐2 cells by using the Lipofectamine 3000 reagent (Life Technologies, USA) following the manufacturer's instructions. Fluorescent dye‐labelled siRNA (GenePharma, China) was adopted to evaluate transfection efficiency, and a scrambled siRNA (NC: 5ʹ‐UUCUCCGAACGUGUCACGUTT‐3ʹ) was used as negative control. Then, the TCam‐2 cells were incubated at 37°C and 5% CO_2_. After 48 hours, the efficiency of the RNAi was evaluated by quantitative real‐time PCR (qRT‐PCR) and Western blot.

### 2.5 Total RNA extraction, RT and qRT‐PCR analysis

The TCam‐2 cells transfected with CSE1L and negative control siRNA were lysed with TRIzol reagent (Invitrogen, Carlsbad, CA, USA), and the total RNA was recovered in accordance with the manufacturer's protocols. Total RNA (1 μg) was reverse‐transcribed using a PrimeScript RT Reagent Kit (Takara, Japan) to obtain cDNA. Then, real‐time PCR was performed with a LightCycler 96 real‐time fluorescent quantitative PCR instrument (Roche, Basel, Switzerland). All qPCR experiments were conducted with RNA extracts from three independent batches of cells, and each reaction was run in triplicate. The expression of *Gapdh* was used as the reference to calculate for the relative expression of *CSE1L* using the 2^−(∆∆ Ct)^ method. The sequence information of the primers was as follows: *CSE1L*‐F 5ʹ‐TTTTGAGTTACCCGAAGA‐3ʹ, *CSE1L*‐R 5ʹ‐TTGTGAAGTGACTGTGCC‐3ʹ, *GAPDH*‐F 5ʹ‐GTCAGCCGCATCTTCTTTTG‐3ʹ and *GAPDH*‐R 5ʹ‐GCGCCCAATACGACCAAATC‐3ʹ.

### 2.6 Protein extraction

Cells were lysed with RIPA lysis buffer (Beyotime, Shanghai, China) supplemented with proteinase inhibitors (Sigma). After centrifugation with 14 000 *g* for 10 minutea at 4°C, the supernatant was collected. The protein concentration of the supernatant was evaluated using a BCA Protein Assay Kit (Pierce, Bonn, Germany). Then, the supernatant was added with SDS loading buffer and boiled at 100°C for 7 minutes. Afterwards, protein samples were stored at −80°C for immunoblotting.

### 2.7 Immunoblotting

Protein samples were loaded onto an 8% separation gel and 5% spacer gel, and the gels were transferred to PVDF membranes (Minipore, Milford, MA, USA). The membranes containing target proteins were blocked with 5% skimmed milk in TBST for 1 hour and probed with rabbit anti‐β‐actin antibody (1:500; Proteintech, Wuhan, China) and rabbit anti‐CSE1L antibody (1:2000; Novus, Littleton, CO, USA) diluted in TBST overnight at 4°C. Then, the membranes were incubated with anti‐rabbit IgG, HRP‐linked secondary antibody (1:10000; Sigma) for 2 hours, and then washed again three times. We visualized protein bands using the ECL Chemiluminescent Substrate Reagent Kit (Invitrogen) and ChemiDoc Chemiluminescent Imaging Analysis System (Bio‐Rad, Hercules, CA, USA).

### 2.8 Cell proliferation experiment

Cell Counting Kit‐8 (CCK‐8; Beyotime) was used to evaluate the growth rate of treated cells. In brief, cells transfected with siRNA were seeded to 96‐well plates at a density of 1000‐2000 cells per well, and the cells were cultured for 6 hours for cell attachment. Then, we cultured the cells for another 0, 12, 24, 36, 48 and 60 hours (10 replicates for each group). CCK‐8 solution (10 μL) was added to each well, and the plates were incubated for 1 hour at 37°C with 5% CO_2_. Thereafter, we used the Synergy HTX Multi‐Mode Microplate Reader (BioTek, Winooski, VT, USA) to determine the absorbance at 450 nm, with a 450‐490 nm detection wavelength. We calculated for the difference and constructed the growth curves of cells using GraphPad Prism 6 (GraphPad‐Prism Software Inc., San Diego, CA, USA).

### 2.9 Cell cycle assay

TCam‐2 cells cultured in six‐well plates were transfected with siRNAs at 30%‐50% confluence and then cultured for 48 hours in 37°C with 5% CO_2_ and DMEM supplemented with 10% FBS. Then, the cells were digested with 0.25% trypsin (Gibco) and fixed in 50% ethanol at −20°C overnight. The cells were incubated with RNase A (Fermentas, Hanover, MD, USA) at 37°C water bath for 30 minutes, stained with 50 μg/mL propidium iodide (Kaiji, Nanjing, China), and then 2 × 10^4^ cells were analysed using a FACSort flow cytometer (BD Biosciences, San Jose, CA, USA). The results were examined using the ModFit LT software (BD Biosciences). Assays were independently performed three times.

### 2.10 Bromodeoxyuridine (BrdU) incorporation assay

TCam‐2 cells were seeded onto coverslips in 12‐well culture plates and transfected with negative control siRNA and *CSE1L*‐siRNA at 30%‐50% confluence. After transfection, the cells were cultured with DMEM supplemented with 10% FBS for 24 hours. The cells were starved in serum‐free DMEM for 16 hours, and 30 μg/mL of BrdU (Abcam) was added to the medium. After 18 hours of culture, the cell slides were fixed with 4% PFA for 15 minutes and washed three times with PBS. The immunocytochemical staining was performed using rat anti‐BrdU antibody (1:500; Abcam) and FITC‐labelled donkey anti‐rat IgG (H + L; 1:200; ZSGB‐BIO). The percentages of BrdU‐positive cells were counted for every 500 cells, and the data were presented from three independent experiments.

### 2.11 Statistical analysis

All quantitative data represent the means and SD of at least three independent experiments. Statistical analyses between different groups were performed using a one‐way ANOVA (**P < 0.01 and *P < 0.05).

## 3 RESULTS

### 3.1 CSE1L is significantly enriched in human seminoma tissues

**Figure 1:**
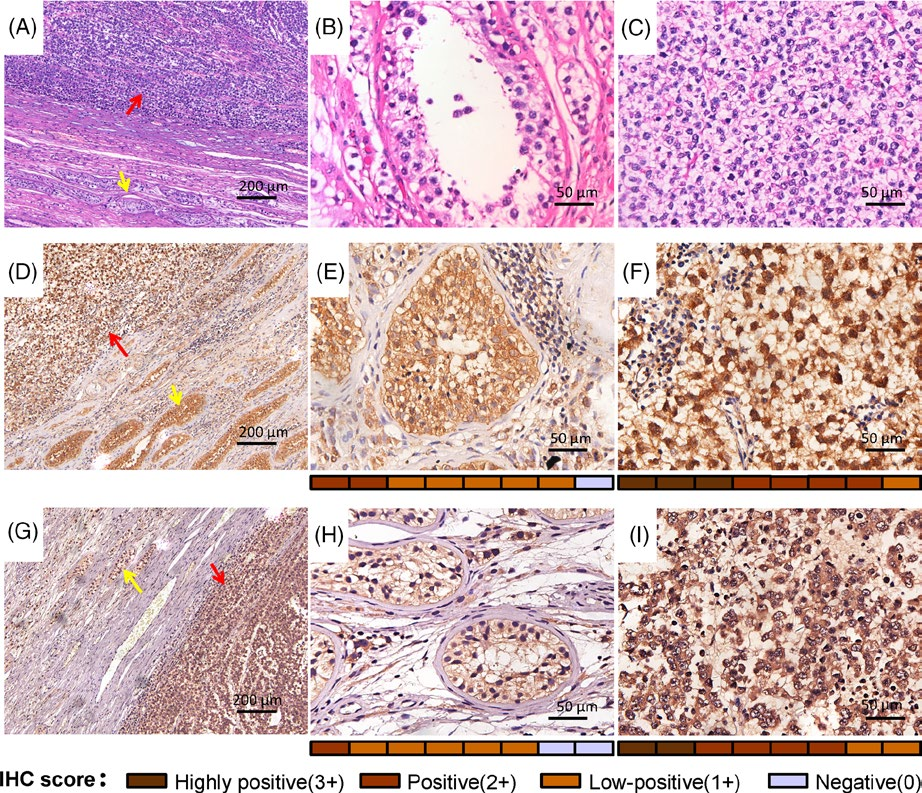
CSE1L is enriched in human seminoma samples in protein level. HE staining image containing human seminoma tissue and testis tissue near the seminoma tissue under a microscope at 100‐ (A) and 400‐fold magnifications of the testis tissue (B) and seminoma tissue (C). Immunohistochemical staining of the testis and seminoma tissue from two patients under 100‐fold magnification (D and G). Immunohistochemistry results of 400‐fold microscopy of CSE1L expression in seminoma (F and I) and normal testis tissues (E and H). Red rows show the seminoma tissue. Yellow rows reveal the seminiferous tubules of the testis tissue near the seminoma. IHC score (0‐3+) for each sample (n = 8) is exhibited as a heat map in the bottom of the 400‐fold represented micrograph

CSE1L is significantly overexpressed in multiple types of tumours in comparison with their corresponding normal tissues, such as the colorectum, liver, breast, thyroid and ovary.^18^, ^19^, ^24^, ^32^, ^33^, ^34^ However, whether CSE1L plays a role in seminomas is unclear. To help diagnose the type of testicular cancers, we first performed HE staining on the seminoma tissues and the corresponding non‐tumour normal tissues from testicular cancer patients (Figure 1A). In the normal testicular tissues, the histomorphology of the seminiferous tubules was unaffected, and different sizes of spermatogenic cells were observed (Figure 1B). The testicular cancer tissues exhibited a typical seminoma morphology. The consistently sized tumour cells were round with a transparent cytoplasm, distinct cell membrane, slightly oval‐shaped nucleus and linear column or evenly distributed chromatin particles (Figure 1C). To further determine and compare the CSE1L expression levels between the normal and seminoma tissues, we performed immunochemical analysis in the samples containing both seminoma and non‐tumour normal tissues (Figure 1D,G). As shown in Figure 1E,H, CSE1L was mainly distributed in the seminiferous tubules and sporadically in the interstitial tissue. By contrast, in the seminoma tissues, CSE1L was intensely expressed in the nuclear and cytoplasmic areas of the tumour cells (Figure 1F,I). Semi‐quantitative scores of CSE1L expression in IHC slides showed a dramatic upregulation of CSE1L in the seminoma samples. These data suggest that the CSE1L protein is upregulated in seminoma tissues and may play an important function in testicular cancer development.

**Figure 2:**
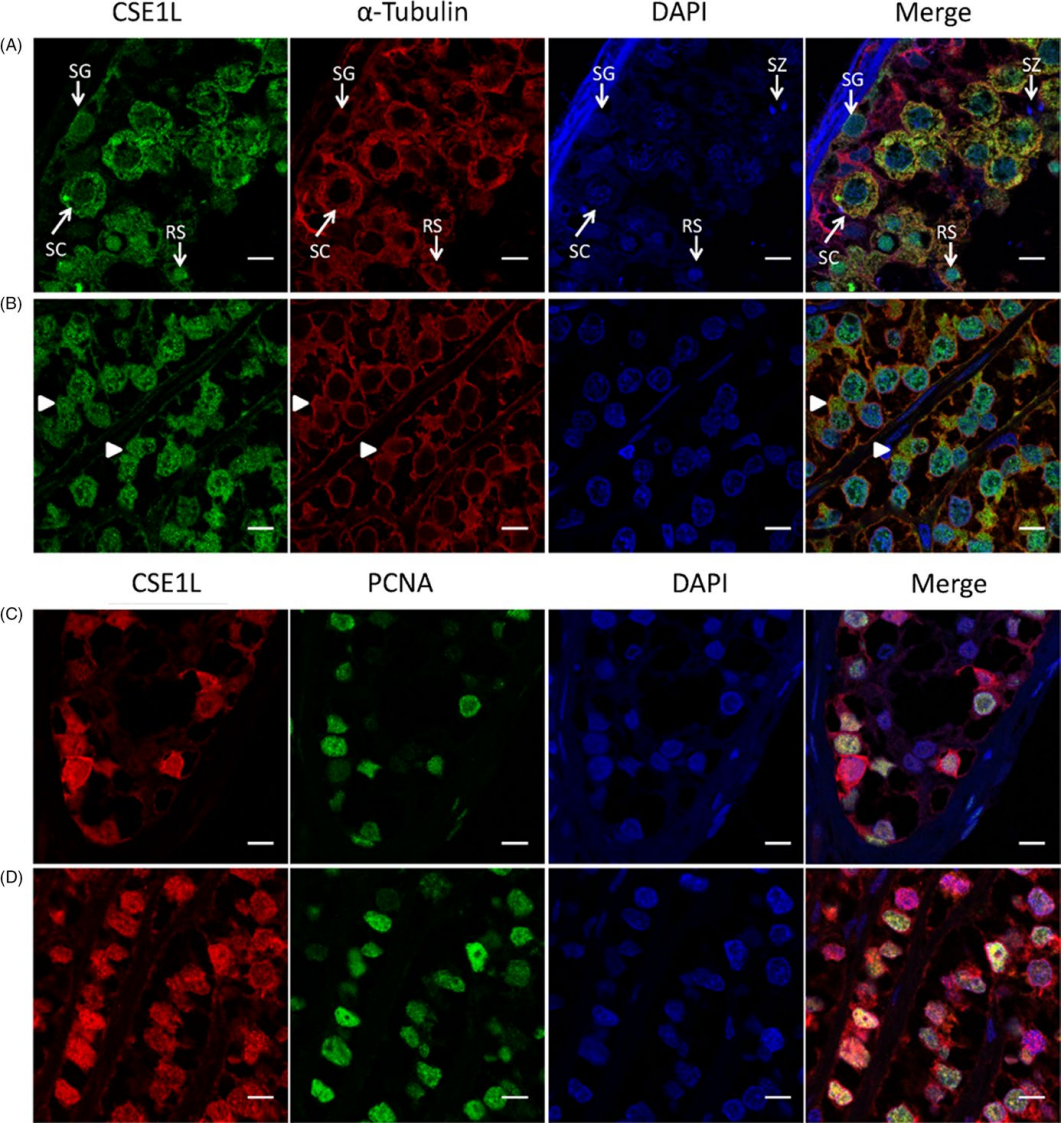
CSE1L is associated with cell proliferation. A, Fluorescence distribution of CSE1L in the seminiferous tubules of the testis tissue. Red rows show CSE1L is co‐localized with α‐tubulin in the cytoplasm of the spermatocytes. White rows exhibit increased nuclear localization of CSE1L was observed in the round spermatids. The different cell types in the testicular seminiferous tubules were labelled. SG, spermatogonia; SC, spermatocyte; RS, round spermatid; SZ, spermatozoa. B, Immunofluorescence images of CSE1L and α‐tubulin in the seminoma cancer cells. White rows indicate the cell enrichment of CSE1L and α‐tubulin in the cytoplasm. C, Localization of CSE1L and PCNA in the testicular seminiferous tubules. D, Expression of CSE1L and PCNA in the seminoma tissue. Scale bar, 8 μm

To further investigate CSE1L's distribution in testicular and seminoma tissues, immunofluorescence staining with α‐tubulin and CSE1L was conducted. CSE1L was mainly distributed in the cytoplasm of the spermatocyte but in the nuclei of spermatogonia and round spermatids (Figure 2A). However, no CSE1L expression was noted in the spermatozoa. CSE1L was also co‐localized with α‐tubulin in the cytoplasm of spermatogenic cells, especially in spermatocytes. These data indicate that CSE1L may function in cell division. In the seminoma tissues, CSE1L was expressed in the nucleus and cytoplasm of most tumour cells, but the CSE1L distribution also tended to concentrate in the cytoplasm of some cells and co‐localized with α‐tubulin, as indicated by the white arrowheads (Figure 2B). In addition, proliferating cell nuclear antigen (PCNA) was expressed in most cells expressing CSE1L in the testis and seminoma tissues (Figure 2C,D), indicating that CSE1L may be closely related to cell proliferation. Overall, we presumed that CSE1L may be highly involved in cell division of seminomas.

### 3.2 Localization of CSE1L during the cell cycle

**Figure 3:**
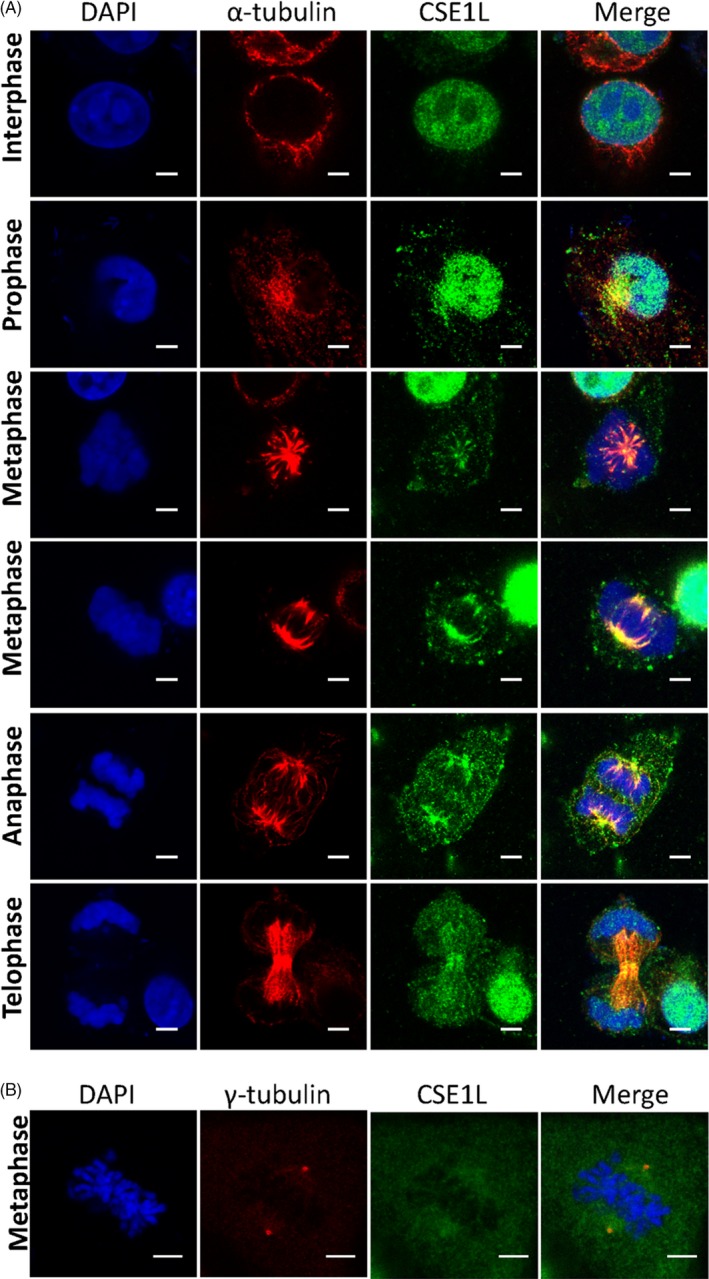
CSE1L protein is co‐localized with spindle during TCam‐2 cell division. A, CSE1L is located in the nucleus in interphase and began to bind with α‐tubulin in the cytoplasm at prophase. During metaphase and anaphase, CSE1L binds along the spindle and some spreads in the cytoplasm. Later in the telophase, CSE1L associates with the spindle fibres and the contractile ring. B, CSE1L co‐localizes with the centrosome at metaphase in TCam‐2 cells. Scale bar, 5 μm

To explore how CSE1L functions in seminoma cancers, we used the seminoma TCam‐2 cells to determine the location of endogenous CSE1L during cell division by immunofluorescence. In the interphase, CSE1L was mainly distributed in the nucleus, whereas α‐tubulin was located in the cytoplasm (Figure 3A). However, in the prophase, besides distributing in the nucleus, the CSE1L protein appeared in the cytoplasm and co‐localized with α‐tubulin. Then, CSE1L in the nucleus disappeared with the depolymerization of the cell nucleus and started to associate with the spindle during metaphase and anaphase. The CSE1L protein was also observed in the cell cortex during the cell cycle. Finally, the CSE1L signal was recovered in the nucleus and found at around the spindle and contractile ring in telophase. CSE1L seemed to be associated with the centrosome in the anaphase; thus, we performed the immunostaining of TCam‐2 cells with CSE1L and γ‐tubulin antibody. As shown in Figure 3B, CSE1L was co‐localized with the centrosome marker γ‐tubulin. These data suggest that CSE1L may serve a special role in the spindle formation of TCam‐2 cells.

### 3.3 Loss of function of CSE1L affects cell proliferation and cell cycle

**Figure 4:**
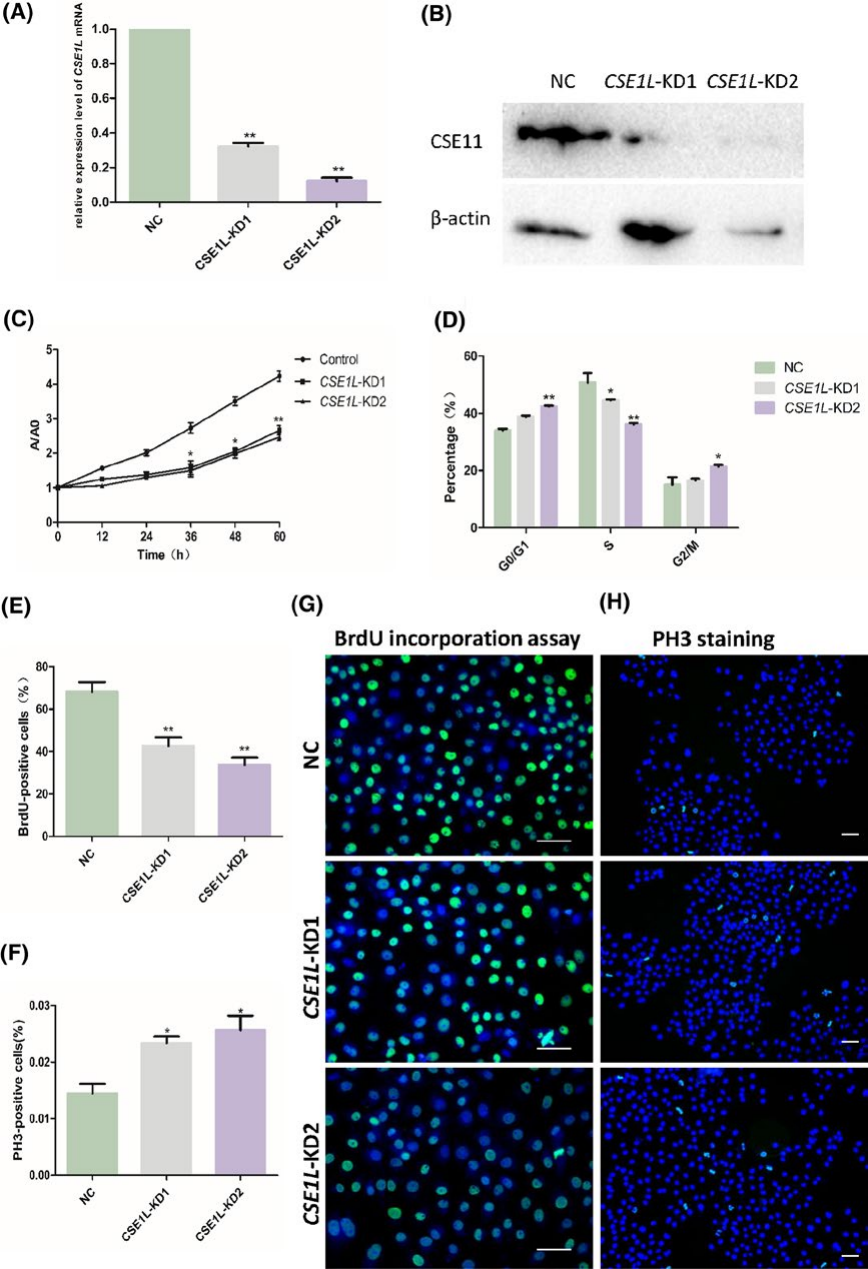
Knockdown of endogenous *CSE1L* inhibits cell proliferation and leads to cell cycle arrest. (A and B) The *CSE1L* knockdown efficiency was examined in TCam‐2 cells. The mRNA (A) and protein (B) levels of *CSE1L* obviously decreased in the TCam‐2 cells transfected with *CSE1L*‐KD1 and *CSE1L*‐KD2 siRNAs. C, Relative proliferation analysis of *CSE1L*‐knockdown groups vs the negative control group by CCK‐8 assay in TCam‐2 cells. Ten independent experiments were performed at each time point in each group. D, The chart shows the cell cycle data obtained by flow cytometry analysis in the NC,* CSE1L*‐KD1 and *CSE1L*‐KD2 groups. The experiments in each group were conducted in three repetitions. (E and G) BrdU incorporation assay was performed in the NC, *CSE1L*‐KD1 and *CSE1L*‐KD2 groups to determine the influence of *Cse1l* knockdown on the growth of TCam‐2 cells. Representative immunofluorescence images and diagram showing the ratio of BrdU‐positive cells in three groups exhibited in G and E, respectively. (F and H) Influence of *CSE1L* knockdown on M phase progression as determined by PH3 staining. The ratio of PH3‐positive cells in three groups was achieved in F and representative images shown in H. Scale bar, 50 μm

Because CSE1L was demonstrated to be associated with the spindle and centrosome in TCam‐2 cells, we opted to interfere with the endogenous *CSE1L* expression by RNAi to investigate the function of CSE1L in regulating the growth of TCam‐2 cells. We synthesized three different siRNAs to knock down *CSE1L*expression. As shown in Figure 4A, compared with the negative control, *CSE1L*‐KD1 and *CSE1L*‐KD2 effectively knocked down the *CSE1L*mRNA levels by 68% and 89%, respectively. The CSE1L protein expression in the TCam‐2 cells transfected with *CSE1L*‐KD1 and *CSE1L*‐KD2 was also significantly diminished relative to that in the control siRNA. This result verifies the knockdown efficiency of the two CSE1L siRNAs. Accordingly, we chose *CSE1L*‐KD1 and *CSE1L*‐KD2 to explore CSE1L function in the TCam‐2 cells.

Next, we inspected the effect of CSE1L deficiency on the proliferation and DNA synthesis of TCam‐2 cells. CCK‐8 results showed that the cell numbers were significantly reduced in the *CSE1L*‐KD1 and *CSE1L*‐KD2 groups than in the negative control at 36, 48 and 60 hours after transfection (Figure 4C). To further investigate the mechanism underlying cell proliferation suppression, we first evaluated the influence of *CSE1L*knockdown on cell cycle progression by flow cytometric analysis. As shown in Figure 4D, the percentage of cells was significantly increased in the G0/G1 phase and decreased in the S phase in the *CSE1L*‐KD1 and *CSE1L*‐KD2 groups, whereas a moderate rise in cell numbers was noted in the G2/M phase in the *CSE1L*‐KD2 group. These results indicate that CSE1L deficiency can induce G1/G0 arrest and delay the G2/M phase of TCam‐2 cells. To verify this result, we continued to evaluate the influence of *CSE1L* knockdown on the DNA synthesis of TCam‐2 cells by using the BrdU incorporation assay. BrdU can incorporate into replicating DNA molecules instead of thymine in proliferation cells; hence, BrdU marked cells in the S phase. As shown in Figure 4E,G, the percentage of BrdU‐positive cells in the *CSE1L*‐KD1 and *CSE1L*‐KD2 groups were obviously decreased relative to that in the control group. Next, we measured cells by PH3, a marker for M phase, to determine whether the suppression of cell growth was partly due to the M phase delay in CSE1L‐deficient cells. Compared with the control group, the *CSE1L*‐KD1 and *CSE1L*‐KD2 groups showed moderately increased numbers of PH3‐positive cells (Figure 4F,H). These data revealed that the loss of function of CSE1L caused the TCam‐2 cells to arrest in the G0/G1 phase, slight delay in the M phase, and become inhibited in cell proliferation.

### 3.4 CSE1L defects in TCam‐2 cells disrupt cell mitosis

**Figure 5:**
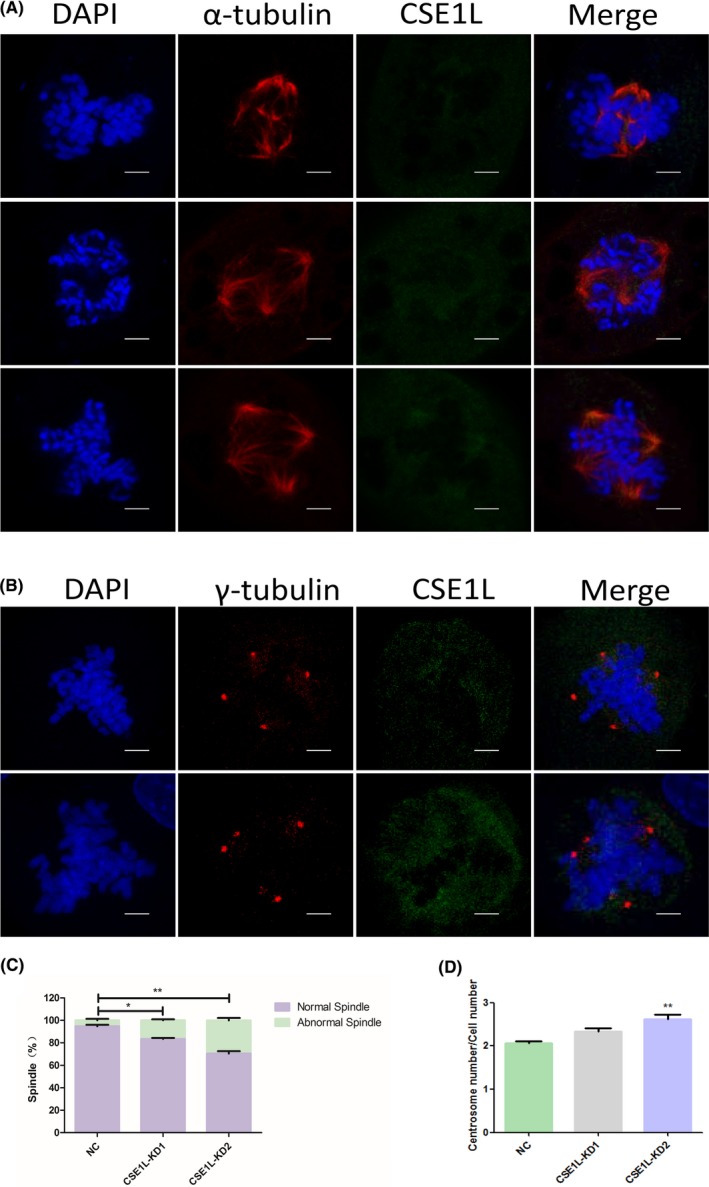
Loss of function of CSE1L disrupts TCam‐2 cell division. A, Immunofluorescent staining images reveal spindles with abnormal geometry and multipolar spindles in *Cse1l*‐deficient TCam‐2 cells. Scale bar, 5 μm. B, Immunofluorescent staining images of dividing cells with four centrosomes but normal dividing cells with two centrosomes. Scale bar, 5 μm. C, The diagram shows the increased ratio of abnormal spindles after *CSE1L* knockdown. D, The graph displays a decrease in *CSE1L* that results in the amplification of centrosomes per cell. The data statistics for C and D were obtained from three replicates for each group, and each replicate was calculated for the number of spindles and centrosomes from 50 dividing cells

In a previous study, we found that CSE1L co‐localized with spindle and centrosomes during TCam‐2 cell division, and CSE1L deficiency induced cell cycle arrest in the G2/M phase; thus, we speculated that CSE1L may disrupt the progression of cell division. To confirm our hypothesis, we performed immunostainings of α‐tubulin and CSE1L in TCam‐2 cells after *Cse1l* knockdown. We found a significant number of mitotic cells with abnormal spindle geometry in the *CSE1L*‐KD1 and *CSE1L*‐KD2 groups compared with those in the TCam‐2 cells transfected with control siRNA. Some abnormal cells were observed with the spindle poles not lying perpendicular to the metaphase plate, and other types of abnormally dividing cells with multipolar spindles frequently occurred in the CSE1L‐deficient cells (Figure 5A). By calculating for the normal and abnormal spindle numbers in the dividing TCam‐2 cells of the NC, *CSE1L*‐KD1 and *CSE1L*‐KD2 groups, we found a rise in the ratio of the dividing cells with abnormal spindles. We also used the γ‐tubulin antibody to assess the centrosome numbers in *CSE1L*‐knockdown cells. As shown in Figure 5B, many mitotic cells showed more than two centrosomes. Statistical data revealed that after *CSE1L* knockdown, the ratio of centrosome number to dividing cell number was higher than that of the normal dividing cells containing two centrosomes (Figure 5D). Overall, these data demonstrated that CSE1L can facilitate the completion of normal cell mitosis in TCam‐2 cells.

## 4 DISCUSSION

The high expression of CSE1L in various types of tumours was reported in many studies. However, no study is available concerning CSE1L expression in TGCTs. Herein, we found that CSE1L was significantly enriched in seminoma than in non‐tumour normal testis tissues, and this result indicated that CSE1L may play a pivotal role in seminoma tumorigenesis. CSE1L was silenced in the seminoma cell line TCam‐2, and the cell growth was inhibited. This result agrees with those of previous studies in other cancer cells. Studies in colorectal cancer cell lines (HCT116, SW480, HT29 and RKO) found that cell proliferation limited by the RNAi of CSE1L and CSE1L‐deficient cells seemed to arrest the G0/G1 phase.^35^, ^36^ The depletion of CSE1L by RNAi in primary papillary thyroid carcinoma cell line B‐CPAP led to reduced cell proliferation; this phenotype was confirmed by the significantly reduced BrdU incorporation in CSE1L knockdown groups.^24^ To investigate the functional significance of CSE1L in osteosarcoma, authors performed the RNAi of CSE1L in the two osteosarcoma cell lines MNNG/HOS and U2OS, and cell growth was significantly inhibited. Cell cycle assay showed a delayed G1 phase transition.^29^ In our study, the cell ratio was increased in the G0/G1 phase and decreased in the S phase in CSE1L‐deficient TCam‐2 cells. The BrdU incorporation in the *CSE1L*‐KD1 and *CSE1L*‐KD2 groups was significantly reduced relative to those in the control groups. Because BrdU is a marker of S phase cells, the above‐mentioned data partly explained the inhibited cell proliferation, which was caused by the delay of cell cycle progression by G1 phase transition suppression.

In our study, we found that the CSE1L protein was clearly co‐localized with spindle during the cell mitosis by immunostaining of CSE1L and α‐tubulin antibodies simultaneously in the TCam‐2 cells. A previous study performed immunofluorescence with anti‐CSE1L and anti‐tubulin antibodies separately in MCF‐7 cells and found that CSE1L was distributed in a pattern similar to tubulin in the dividing cells^37^; however, they have not shown a co‐localization of CSE1L with tubulins in mitotic cells. Tai et al^38^ demonstrated that CSE1L can bind to α‐ and β‐tubulin and enhance the assembly of microtubules in the cytoplasm of the MCF‐7 cells. We found that CSE1L was mainly localized in the nucleus in the interphase of TCam‐2 cells, whereas in MCF‐7 cells, CSE1L was exclusively distributed in the cytoplasm and co‐localized with microtubule.^38^ This difference may be attributed to cell type diversity. When CSE1L expression was disrupted in TCam‐2 cells, spindle formation was seriously influenced, and the chromosome exhibited a disordered alignment. By cell cycle assay, the cell ratio in the G2/M phase also exhibited a moderately increasing trend in CSE1L‐deficient cells, and PH3 immunostaining verified an increase in M phase cells by CSE1L knockdown. This phenotype was observed in other microtubule‐ and spindle‐associated proteins, such as BuGZ. The RNAi of BuGZ in several human cell lines resulted in chromosomal misalignment and mitotic block in the M phase.^39^ KIFC3 and KIF3b, members of the human kinesin family, have been reported to be highly expressed in seminoma tissues; the proteins also bind along the spindle and affect spindle formation in Hela cells.^40^, ^41^ Given these data, we conclude that CSE1L is essential to proper cell division and the disrupted CSE1L expression in TCam‐2 cells led to M phase delay.

Centrosomes play an important role in orderly chromosome segregation by contributing to bipolar spindle formation during cell mitosis.^42^ The mitotic spindle of a normal cell possesses two poles, each containing a single centrosome.^43^ However, most cancer cells harbour redundant centrosomes through the process termed centrosome amplification.^44^ This characteristic is expected to cause cells to form multipole spindles and disrupt cell mitosis.^45^ Cancer cells settle this problem by clustering extra centrosomes into two poles to form a pseudo‐bipolar spindle.^46^ Several proteins distributed in the centrosome have been shown to be involved in regulating centrosomal gathering.^47^, ^48^ In the TCam‐2 cells, the CSE1L protein co‐localizes with the centrosomal marker γ‐tubulin; thus, we presume that CSE1L is engaged in centrosomal clustering in cancer cells. However, this assumption requires accurate experiments for verification.

## CONFLICT OF INTEREST

The authors declare no conflict of interests for this article.
